# Proteomic Insights into Effects of a Camel Milk-Derived Peptide on Insulin Resistance: Modulation of Metabolic, Oxidative, and Signaling Pathways

**DOI:** 10.3390/foods15071177

**Published:** 2026-04-01

**Authors:** Issoufou Katambe Mohamed, Yufei Hua, Xiangzhen Kong, Xingfei Li, Yeming Chen, Caimeng Zhang, Mouhamed Fall, Abuubakar Hassan Ramadhan

**Affiliations:** 1 State Key Laboratory of Food Science and Technology, Jiangnan University, 1800 Lihu Avenue, Wuxi 214122, China; mikatambe@yahoo.fr (I.K.M.); xzkong@jiangnan.edu.cn (X.K.); lixingfeiangel@163.com (X.L.); cmzhang@jiangnan.edu.cn (C.Z.); 2 School of Food Science and Technology, Jiangnan University, Wuxi 214122, China; 3 Institut National de la Recherche Agronomique du Niger, Niamey 429, Niger

**Keywords:** insulin resistance, TYYPPQ, proteomics, insulin signaling pathways, mitochondrial function

## Abstract

Insulin resistance is a multifactorial cellular state involving coordinated alterations in protein homeostasis and organelle function; however, its proteome-wide organization and response to bioactive peptides remain incompletely defined. In this study, we employed DIA-based quantitative proteomics to characterize global protein abundance changes associated with insulin resistance in HepG2 cells and to examine proteomic remodeling following treatment with a camel milk-derived peptide (P2). Comparative proteomic profiling revealed that insulin-resistant cells exhibit extensive reorganization of protein networks linked to redox regulation, endoplasmic reticulum protein processing, mitochondrial metabolism, lysosomal function, and extracellular matrix-associated components. Gene Ontology, KEGG pathway, protein domain, and subcellular localization enrichment analyses consistently indicated disruption of organelle-associated proteomic architecture rather than isolated pathway perturbations. Peptide TYYPPQ treatment was associated with selective, rather than global, proteomic shifts, prominently affecting mitochondrial and peroxisome-associated protein groups as well as extracellular and secretory proteins. Enrichment and localization analyses suggest that peptide exposure reshapes organelle-linked protein representation patterns without implying direct activation of signaling pathways or physiological restoration. Collectively, these results define insulin resistance and peptide responsiveness at a systems-level proteomic resolution and establish an organelle-resolved framework for interpreting peptide-induced proteomic remodeling in insulin-resistant hepatocyte models. This dataset provides a foundation for future targeted functional validation of candidate pathways identified through proteomic association.

## 1. Introduction

Type 2 diabetes mellitus (T2DM) is a chronic metabolic disorder characterized by insulin resistance (IR), in which peripheral tissues fail to respond adequately to insulin, leading to impaired glucose uptake, persistent hyperglycemia, and progressive metabolic dysfunction. At the molecular level, defective activation of the insulin receptor (IR) and disruption of its downstream signaling cascades including the PI3K–Akt and AMP-activated protein kinase (AMPK) pathways are central to the development of IR, contributing to altered glucose transport, lipid accumulation, and chronic inflammation [1,2,3]. Restoring IR signaling remains a primary therapeutic goal in T2DM [4,5], yet many small-molecule insulin sensitizers suffer from limited specificity or adverse effects.

In recent years, bioactive food-derived peptides have emerged as a promising class of insulin-sensitizing agents [6] due to their high biocompatibility, low toxicity, and capacity to modulate multiple metabolic targets. Several milk-derived peptides, particularly from bovine and camel milk, have been reported to stimulate insulin signaling pathway [7]. Camel milk is of particular interest because of its unique protein composition and high abundance of bioactive peptide precursors, which have been linked to anti-diabetic, anti-glycation, and insulin-sensitizing activities [8]. However, despite growing evidence that camel milk hydrolysates improve glycemic control, the molecular mechanisms by which individual camel milk peptides regulate insulin receptor signaling remain poorly defined.

Peptide-mediated modulation of the insulin receptor represents an especially attractive strategy, as it allows for selective tuning of receptor conformation and kinase activity through non-competitive or allosteric interactions. Recent studies have demonstrated that short bioactive peptides can bind to regulatory regions of kinases, stabilize activation loops, or influence phosphorylation dynamics [9], thereby amplifying downstream signaling without competing with endogenous ligands. These properties make peptides particularly suitable for correcting the subtle signaling defects that underlie insulin resistance.

In our previous work [9], we identified a camel milk-derived hexapeptide, TYYPPQ, as a potent modulator of insulin signaling in insulin-resistant HepG2 cells. TYYPPQ activated the insulin receptor through a non-competitive allosteric mechanism, binding within the IR kinase domain and stabilizing its activation loop, which in turn promoted Akt phosphorylation and glucose-regulatory signaling. Structure-based modeling, including molecular docking, molecular dynamics (MD), and in silico mutagenesis, revealed persistent hydrogen bonding with Glu1047, a conserved catalytic residue of the insulin receptor kinase domain that is positioned near the activation-loop tyrosines Tyr1158, Tyr1162, and Tyr1163. This interaction is mediated by the peptide backbone amide –NH– within the –C(=O)–NH– moiety adjacent to the aromatic ring and thioether group, forming a stable anchoring contact within the catalytic cleft. In parallel, favorable interactions with residues of the DFG motif further stabilize the active kinase conformation, collectively promoting receptor autophosphorylation and enhanced insulin receptor activation.

Although these findings established TYYPPQ as a direct molecular activator of the insulin receptor, they primarily addressed receptor-level interactions and signaling initiation. Insulin resistance, however, is a systems-level disorder involving coordinated alterations in metabolism, oxidative stress, inflammation, protein folding, and intracellular trafficking. Whether peptide-mediated IR activation is sufficient to reprogram these downstream cellular processes remains largely unknown, particularly for camel milk-derived peptides.

To address this critical gap, the present study applies DIA-based label-free quantitative proteomics using a UHPLC–Orbitrap Astral (Direct DIA) platform to systematically characterize global protein expression changes induced by TYYPPQ in insulin-resistant HepG2 cells. By integrating principal component analysis (PCA), Gene Ontology (GO), KEGG pathway enrichment, and subcellular localization profiling, we aim to elucidate how TYYPPQ-mediated insulin receptor activation propagates through cellular networks to restore metabolic homeostasis. This systems-level analysis validates the functional consequences of insulin receptor activation by TYYPPQ and identifies key pathways and protein networks involved in its anti-diabetic action.

## 2. Materials and Methods

### 2.1. Peptide Synthesis

Peptide TYYPPQ sequence previously [10] identified from camel milk, was synthesized by Sangon Biotech (Shanghai, China). The synthetic TYYPPQ peptide was purified by reverse-phase HPLC to a purity of 95%, as determined by peak area normalization. Peptide identity was confirmed by LC–MS, which showed a molecular ion consistent with the theoretical monoisotopic mass of TYYPPQ (767.40 Da), and MS/MS fragmentation confirmed the expected sequence. 

### 2.2. Modeling Insulin Resistance in HepG2 Cells and Peptide Intervention

HepG2 cells were seeded in 6-well culture plates and incubated overnight at 37 °C in a humidified atmosphere containing 5% CO_2_ to allow cell adherence. Insulin resistance was induced by exposing the cells to 18 mM glucosamine (S1635, Beyotime Biotechnology, Shanghai, China) in high-glucose DMEM supplemented with 10% fetal bovine serum (FBS) for 16 h. This glucosamine-based model is a well-established and widely used method to impair insulin signaling in hepatocytes, as previously demonstrated in multiple studies showing reduced insulin receptor and PI3K–Akt pathway activity and impaired insulin responsiveness [11,12,13,14], and as recommended by the reagent manufacturer (Beyotime, S1635). Moreover, in our previous study using the same model [9], we confirmed successful induction of insulin resistance by observing a significant reduction in insulin receptor (IR) mRNA expression (0.62-fold vs. control, *p* < 0.05) and decreased insulin-stimulated IR phosphorylation, validating impaired insulin responsiveness.

After induction, cells were gently washed with cold phosphate-buffered saline (PBS) and incubated in high-glucose DMEM containing 10% FBS and 100 µM camel milk-derived peptide TYYPPQ for 24 h. Three experimental groups were established: (1) healthy control HepG2 cells (CON), (2) insulin-resistant HepG2 cells (MOD), and (3) insulin-resistant HepG2 cells treated with TYYPPQ (P2). Each group was prepared in biological triplicate (N = 3), yielding a total of nine samples. Following treatment, cells were immediately harvested for protein extraction and downstream proteomic analysis.

To confirm the establishment of insulin resistance in HepG2 cells, insulin signaling was evaluated by examining Akt phosphorylation status using Western blot analysis. Total cellular proteins (20–30 µg per sample) were separated by SDS–PAGE and transferred onto nitrocellulose membranes. Membranes were blocked for 1 h with 5% BSA or non-fat milk to reduce non-specific binding and subsequently incubated overnight at 4 °C with primary antibodies directed against phosphorylated Akt (Thr308) and total Akt.

Following incubation with appropriate horseradish peroxidase-conjugated secondary antibodies, immunoreactive bands were visualized using an enhanced chemiluminescence detection system. Band intensities were quantified using ImageJ software (1.53c, NIH, Bethesda, MD, USA). Phosphorylated Akt levels were normalized to total Akt expression, while total Akt levels were normalized to β-actin.

### 2.3. Total Protein Extraction

The sample was transferred to a 1.5 mL centrifuge tube and lysed with DB lysis buffer (6 M urea, 100 mM TEAB, pH 8.5), followed by ultrasonication for 5 min in an ice water bath using an ultrasonic probe Ultrasonic Cell Disruptor: Model JY92-11N (Ningbo Xinzhi, Ningbo, Zhejiang Province, China). The lysate was centrifuged at 12,000× *g* for 15 min at 4 °C, and the supernatant was collected. Proteins were reduced by adding 1 M DTT and incubating for 1 h at 56 °C, followed by alkylation with 45 μL of 0.5 M iodoacetamide for 1 h at room temperature in the dark. The reaction was then quenched by placing the samples on ice for 2 min.

### 2.4. Protein Quality Test

BSA standard protein solution was prepared according to the instructions of the Bradford protein quantification kit, with a gradient concentration ranging from 0 to 0.5 g/L. BSA standard protein solutions and sample solutions at different dilutions were added into a 96-well plate to a final volume of 20 µL, with each gradient repeated in triplicate. G250 dye solution (Coomassie Brilliant Blue G250, Solarbio, Beijing, China, Product Code C8420-10g, 10 g/bottle) was quickly added to each well (180 µL), and the plate was incubated at room temperature for 5 min. Absorbance at 595 nm was then measured. The standard curve was plotted using the absorbance of the BSA standards, and the protein concentration of each sample was calculated.

### 2.5. Enzymatic Digestion

Each protein sample was taken and the volume was adjusted to 100 μL with DB lysis buffer (6 M urea, 100 mM TEAB, pH 8.5). Trypsin and 100 mM TEAB buffer were added, and the samples were mixed and digested at 37 °C for 4 h. The urea–TEAB system is a standard proteomics digestion condition, as urea exhibits minimal inhibition of trypsin activity at 37 °C and is widely used for efficient protein denaturation and enzymatic accessibility [15]. Additional trypsin was then added, and digestion was continued overnight. Formic acid was added dropwise to the digested samples, and the pH was monitored using pH indicator strips until it reached below 3. The samples were then centrifuged at 12,000× *g* for 5 min at room temperature. The supernatant was slowly loaded onto a C18 desalting column, washed three times with washing buffer (0.1% formic acid, 3% acetonitrile), and then eluted with elution buffer (0.1% formic acid, 70% acetonitrile). The eluates were collected and lyophilized.

### 2.6. Liquid Chromatography–Tandem Mass Spectrometry (LC–MS/MS)

Mobile phase A consisted of 100% water containing 0.1% formic acid, and mobile phase B consisted of 80% acetonitrile containing 0.1% formic acid. Lyophilized peptide samples were dissolved in 10 µL of solvent A, centrifuged at 14,000× *g* for 20 min at 4 °C, and 200 ng of the supernatant was injected for LC–MS/MS analysis.

Peptides were separated using a Vanquish Neo UHPLC system (Thermo Fisher Scientific, Waltham, MA, USA) equipped with a C18 trap column (5 mm × 300 µm, 5 µm; Thermo Fisher) maintained at 50 °C, followed by a PepMap™ Neo analytical C18 column (150 µm × 15 cm, 2 µm; Thermo Fisher).

Mass spectrometry was performed on a Thermo Orbitrap Astral mass spectrometer equipped with an Easy-Spray ESI source (Thermo Fisher, Bremen, Germany). The spray voltage was set to 2.0 kV and the ion transfer tube temperature to 290 °C. Data were acquired in data-independent acquisition (DIA) mode with a full MS scan range of *m*/*z* 380–980. The MS1 resolution was 240,000 (at *m*/*z* 200), AGC target was 500%, and the precursor isolation window was 2 Th with 300 DIA windows. Fragment ions were acquired over a range of *m*/*z* 150–2000 with an Astral resolution of 80,000, normalized collision energy (NCE) of 25%, and maximum injection time of 3 ms. Raw data were recorded in Thermo raw format.

### 2.7. MS Data Processing and Protein Identification

Raw MS files were analyzed using DIA-NN software DIA-NN 1.8.1 (DirectDIA mode). Protein identification and quantification were performed against the corresponding protein database. Precursor and fragment mass tolerances were automatically optimized by the software. Carbamidomethylation of cysteine was set as a fixed modification, while N-terminal methionine loss was allowed as a variable modification, with up to two missed cleavages permitted.

Retention time calibration was performed using iRT peptides spiked into each sample. Peptide spectrum matches (PSMs) were filtered to retain only high-confidence identifications with a confidence level ≥ 99%. False discovery rate (FDR) was controlled at 1% at both peptide and protein levels, and precursor ion q-value cutoff was set to 0.01.

### 2.8. The Functional Analysis of Protein and DEP

Gene Ontology (GO) and InterPro (IPR) functional analysis were conducted using the interproscan program interproscan-5.22-61.0 against the non-redundant protein database (including Pfam, PRINTS, ProDom, SMART, ProSite, PANTHER) [16], and the databases of COG (Clusters of Orthologous Groups) and KEGG (Kyoto Encyclopedia of Genes and Genomes) were used to analyze the protein family and pathway. DEPs were used for Volcanic map analysis, cluster heat map analysis and enrichment analysis of GO, IPR and KEGG [17].

### 2.9. Statistical Analysis

All experiments were performed with at least three independent biological replicates. DIA data were processed using DIA-NN. Peptide precursors and protein groups were filtered at a 1% false discovery rate (Global.Q.Value < 0.01 and PG.Q.Value < 0.01)

For proteomics analysis, differentially expressed proteins were defined using a fold change threshold of >1.2 for upregulation or <0.83 for downregulation, together with a *p* value < 0.05. Statistical significance was determined using a two-tailed Student’s *t*-test. All statistical analyses were conducted using standard data analysis software, and differences with adjusted *p* < 0.05 were considered statistically significant.

## 3. Results and Discussion

### 3.1. Validation of Insulin Resistance and Peptide Responsiveness in HepG2 Cells via Akt Phosphorylation

**Figure 1:**
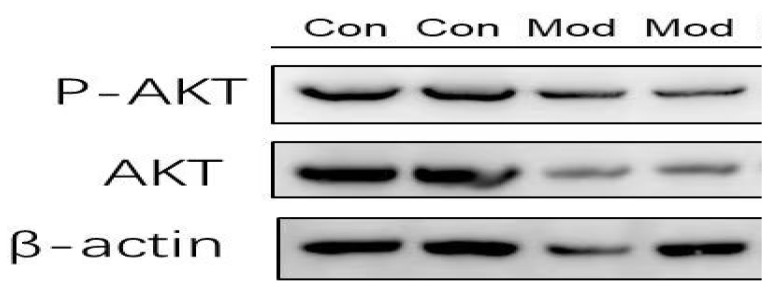
Validation of insulin resistance and of Akt signaling in HepG2 cells. Western blot analysis of phosphorylated Akt (p-Akt), total Akt, and β-actin in control cells and insulin-resistant model cells. p-Akt levels were normalized to total Akt and β-actin was used as a loading control.

To contemporaneously validate the insulin-resistant state of HepG2 cells used in the present study, insulin signaling was assessed by Western blot analysis of Akt phosphorylation. As shown in Figure 1, insulin-resistant cells exhibited a marked reduction in phosphorylated Akt (p-Akt) compared with healthy control cells, while total Akt levels remained relatively unchanged, confirming impaired insulin signaling.

### 3.2. Analysis of Protein Differences: Comparative Analysis of PCA Between Healthy Cells (Control), Insulin Resistant Cells (MOD) and Insulin Resistant Cells Treated with Peptide (P2)

**Figure 2:**
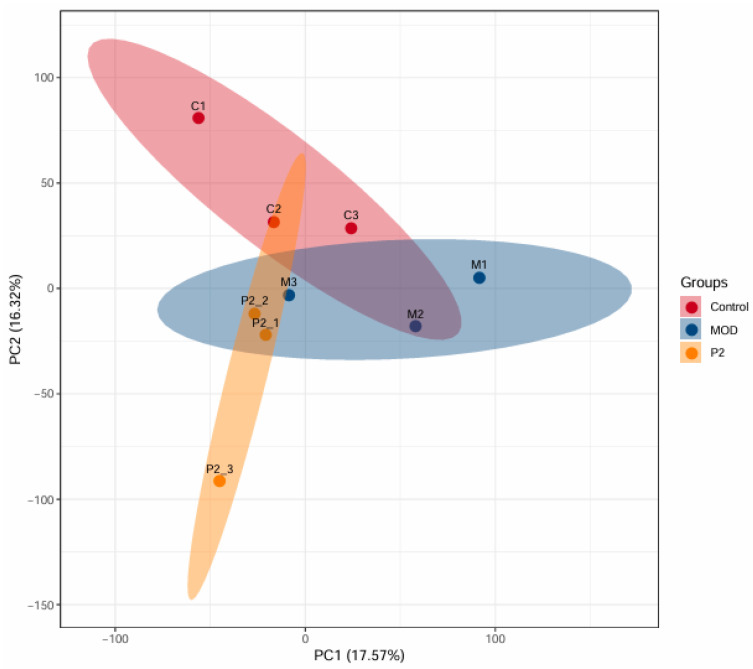
PCA of protein expression profiles between healthy cells (control), insulin resistant cells (MOD) and insulin resistant cells treated with peptide. The abscissa PC1 and ordinate PC2 represent the scores of the first and second principal components, respectively, and the scatter color indicates the experimental grouping of the sample. Each point corresponds to an individual biological replicate: C1–C3 (healthy control, triplicate), M1–M3 (insulin-resistant model, triplicate), and P2-1–P2-3 (peptide-treated insulin-resistant cells, triplicate). Sample grouping is indicated by color.

In this study, DIA-based quantitative proteomics technology was used, and a total of 7068 proteins were identified across 9 samples (n = 3 per group), all of which were quantitatively analyzed. Proteins with a fold change greater than 1.2 (upregulated) or less than 0.83 (downregulated), and a *p*-value < 0.05, were considered significantly differentially expressed. The Principal Component Analysis (PCA) plot (Figure 2) provides a visual summary of the proteomic variation among three experimental groups: healthy control cells (C, red), insulin-resistant cells (MOD, blue), and insulin-resistant cells treated with the peptide TYYPPQ (P2, orange). The plot clearly illustrates a distinct separation between the groups, with the red, blue, and orange ellipses indicating separate proteomic profiles. This confirms that insulin resistance significantly alters protein expression compared to healthy controls. The P2 group (peptide-treated) does not fully overlap with the control group, indicating that TYYPPQ treatment does not fully restore the proteome to a healthy state. However, the P2 group is also clearly separated from the MOD group, suggesting beneficial changes in protein expression patterns due to the peptide treatment. The separation of P2 from MOD suggests a partial restoration or reprogramming of cellular pathways. Further pathway analysis, including GO enrichment and KEGG pathway analysis, could help identify specific proteins and metabolic networks influenced by the peptide treatment.

### 3.3. Differential Protein Enrichment Analysis

#### 3.3.1. GO Enrichment Analysis Between Healthy Cells and Insulin Resistant Cells

**Figure 3:**
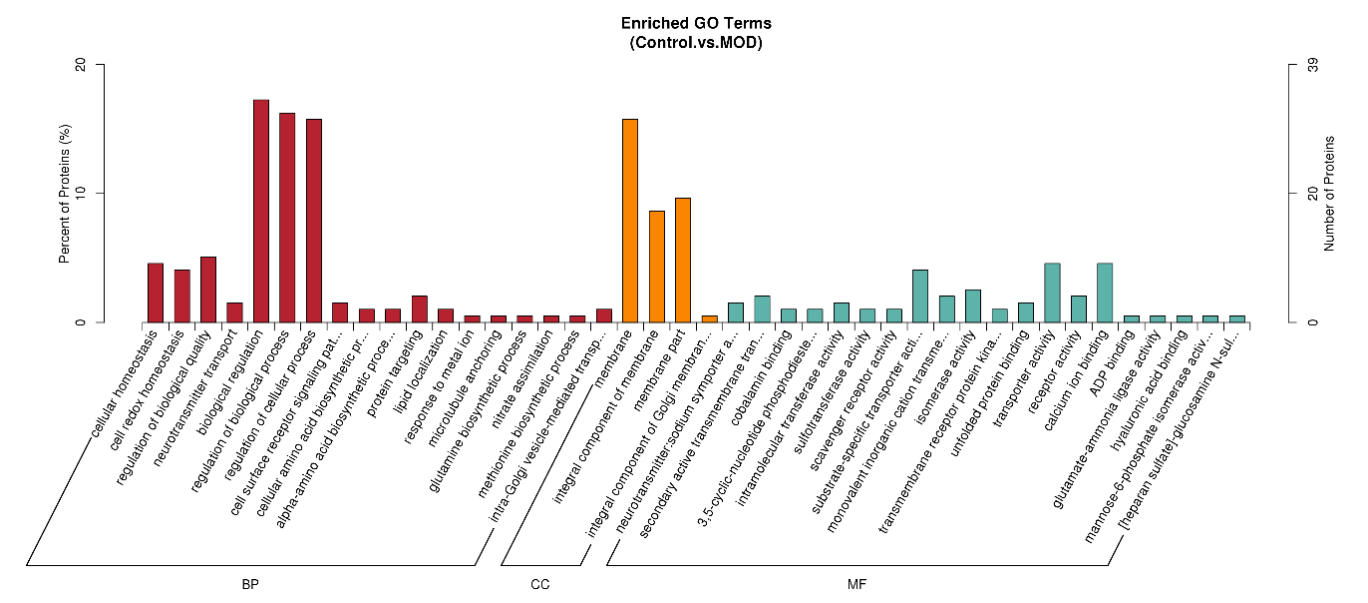
GO enrichment analysis of differentially expressed proteins between healthy cells (Control) and insulin resistant cells (MOD) groups. Gene Ontology (GO) enrichment of significantly differentially expressed proteins (adjusted *p* < 0.05, |log_2_ fold change| ≥ 1) between Control and MOD groups was performed using a GO over-representation analysis with the identified proteome as background. Enriched terms are shown for Biological Process (BP), Cellular Component (CC), and Molecular Function (MF) categories. Bars indicate the percentage of annotated proteins, and only terms with FDR-corrected *p* < 0.05 are displayed. The ellipsis (“…”) in the figure labels indicate truncated terms due to space limitations. The full terms are as follows: BP: cell surface receptor signaling pathway; cellular amino acid biosynthetic process; alpha-amino acid biosynthetic process; CC: integral component of Golgi membrane; MF: neurotransmitter: sodium symporter activity; secondary active transmembrane transporter activity; 3,5-cyclic-nucleotide phosphodiesterase activity; substrate-specific transporter activity; monovalent inorganic cation transmembrane transporter activity; transmembrane receptor protein tyrosine kinase activity; mannose-6-phosphate isomerase activity; [heparan sulfate]-glucosamine N-sulfotransferase activity.

Gene Ontology (GO) enrichment analysis comparing healthy (Con) and insulin-resistant (MOD) cells (Figure 3) revealed that insulin resistance is predominantly associated with alterations in biological processes (BP) related to redox balance, cellular quality control, and regulatory signaling, alongside changes in specific molecular functions (MF) and cellular components (CC). Among BP terms, cellular homeostasis (GO:0019725), a standardized Gene Ontology term defined by the Gene Ontology Consortium, was the most significantly enriched process (adjusted *p* = 0.00456).This enrichment was driven by a tightly connected network of redox-active and protein-folding proteins, including thioredoxin domain-containing proteins (TXNDC9, TXNDC15, TXNDC16), protein disulfide isomerases (PDI, PDIA3, PDIA4, PDIA6), glutaredoxin-1, and ferritin heavy chain. Closely related, cell redox homeostasis (GO:0045454; BP; adjusted *p* = 0.00551) was also significantly enriched and shared nearly identical anchor proteins, highlighting oxidative stress and endoplasmic reticulum (ER) redox imbalance as central features distinguishing insulin-resistant cells from healthy controls.

Consistent with impaired proteostasis, the BP term regulation of biological quality (GO:0065008; adjusted *p* = 0.01663) was enriched, again dominated by thioredoxin- and PDI-family proteins, with the additional contribution of seipin, a regulator of lipid droplet biogenesis and ER integrity. The convergence of these BP terms underscores a coordinated disruption of redox control, protein folding, and cellular quality surveillance mechanisms in insulin-resistant cells, processes known to interfere with insulin signaling and metabolic homeostasis.

In parallel, significant enrichment was observed for transport-related molecular functions (MF) and biological processes (BP). Neurotransmitter: sodium symporter activity (GO:0005328; MF; adjusted *p* = 0.00748) and neurotransmitter transport (GO:0006836; BP; adjusted *p* = 0.01663) were driven by sodium- and chloride-dependent taurine (SLC6A6), creatine (SLC6A8), and glycine (SLC6A9) transporters, indicating altered ion-coupled solute transport in insulin-resistant cells. This was further supported by enrichment of secondary active transmembrane transporter activity (GO:0015291; MF; adjusted *p* = 0.03162), which included the sodium/hydrogen exchanger NHE8, suggesting broader dysregulation of electrochemical gradient-dependent transport that may influence cellular energy balance, osmotic stress, and insulin responsiveness.

At a higher organizational level, insulin resistance was associated with widespread changes in biological regulation (GO:0065007; BP; adjusted *p* = 0.03162) and regulation of biological process (GO:0050789; BP; adjusted *p* = 0.04691). These terms encompassed extensive signaling and regulatory networks involving EGFR, STAT2, AKAP12, CBL, phosphodiesterases (PDE3, PDE4A), Rab and Rho GTPase regulators, Apaf-1, and multiple redox-active proteins. The enrichment of these BP categories reflects global remodeling of signal transduction, apoptosis, cytoskeletal organization, vesicle trafficking, and lipid metabolism in insulin-resistant cells, consistent with systemic metabolic dysfunction.

Beyond BP and MF, enrichment analysis also identified trends at the level of cellular components (CC). The term membrane (GO:0016020; CC; adjusted *p* = 0.05820) included a large cohort of transporters and receptors such as EGFR, Frizzled-7, ABC transporters, SLC6 family transporters, NPC1, and NHE8, indicating that many insulin-resistance-associated proteins localize to cellular and organelle membranes, where they can directly modulate signaling and nutrient transport. Additionally, cobalamin binding (GO:0031419; MF; adjusted *p* = 0.05440), anchored by methylmalonyl-CoA mutase and methionine synthase, suggests a potential link between insulin resistance and altered vitamin B12-dependent mitochondrial and one-carbon metabolism, although this enrichment was comparatively weaker.

Collectively, these GO enrichments indicate that insulin resistance is characterized by coordinated disturbances in redox-related biological processes, membrane-associated transport and signaling functions, and global regulatory networks, with thioredoxin- and protein disulfide isomerase-centered redox systems emerging as dominant anchor nodes. This functional signature provides a mechanistic framework linking oxidative stress, ER dysfunction, and impaired metabolic signaling in insulin-resistant cells.

#### 3.3.2. GO Enrichment Analysis Between Insulin Resistant Cells (MOD) and Insulin Resistant Cells Treated with Peptide (P2)

**Figure 4:**
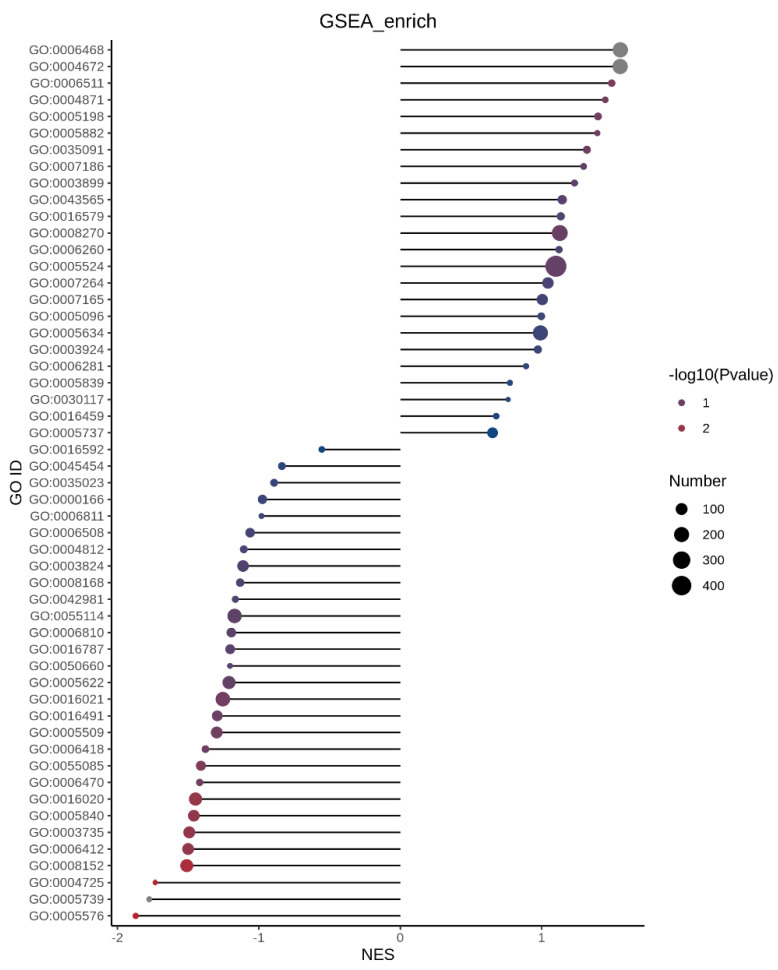
Gene Set Enrichment Analysis (GSEA) of insulin-resistant HepG2 cells treated with the peptide. Gene Set Enrichment Analysis (GSEA) was performed to compare insulin-resistant HepG2 cells with peptide-treated insulin-resistant HepG2 cells using a ranked list of all quantified proteins. Enrichment was assessed against Gene Ontology (GO) gene sets, and results are presented as normalized enrichment scores (NES). Positive NES values indicate gene sets enriched in peptide-treated cells, whereas negative NES values indicate enrichment in untreated insulin-resistant cells. Dot size represents the number of proteins contributing to each GO term, and dot color corresponds to −log10 (*p* value). adjusted *p* ≥ 0.05 are shown as non-significant enrichment trends.

GO over-representation analysis did not reveal statistically significant enrichment of BP, MF, or CC terms following peptide treatment in insulin-resistant cells. This likely reflects the dependence of GO analysis on strict differential expression thresholds, which may overlook subtle yet coordinated proteomic shifts. In contrast, Gene Set Enrichment Analysis (GSEA) (Figure 4), which evaluates ranked changes across the entire proteome without arbitrary cutoffs, revealed significant pathway-level modulation. Gene Ontology (GO) enrichment analysis GSEA comparing insulin-resistant HepG2 cells with those treated with the peptide revealed a significant enrichment in the extracellular region (GO:0005576; adjusted *p*-value = 0.038). This suggests that the peptide may modulate processes related to secretion, extracellular signaling, or interactions within the cellular microenvironment. Notably, proteins associated with this compartment include Insulin-like Growth Factor-Binding Protein 4 (IGFBP4), Serine Protease HTRA1, and Group XIIA Secretory Phospholipase A2, all of which play roles in regulating growth factor availability, extracellular matrix remodeling, and lipid signaling, respectively. Other terms, such as mitochondrion (GO:0005739; adjusted *p*-value = 0.056) and protein tyrosine phosphatase activity (GO:0004725; adjusted *p*-value = 0.058), showed trends toward enrichment but did not reach statistical significance. Collectively, these findings indicate that the peptide’s effects in insulin-resistant cells may be mediated in part through modulation of key extracellular proteins that influence cell signaling and metabolic regulation.

#### 3.3.3. KEGG Enrichment Analysis

**Figure 5:**
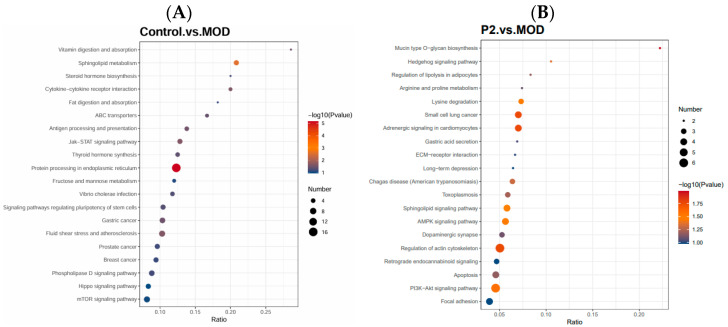
(**A**) KEGG enrichment analysis between Control (healthy cells) and insulin resistant cells (MOD), (**B**) KEGG enrichment analysis between insulin resistant cells (MOD) and insulin resistant cells treated with peptide (P2). The x-axis represents the enrichment ratio of proteins associated with KEGG, while the y-axis lists the enriched functional KEGG pathways. Bubble size indicates the number of proteins contributing to each domain, and color intensity corresponds to −log10 (*p*-value). Statistical significance was assessed using adjusted *p*-values corrected for multiple testing (Benjamini–Hochberg method), with an adjusted *p* < 0.05 considered significant. KEGG pathways with adjusted *p* ≥ 0.05 are shown as non-significant enrichment trends.

As shown in Figure 5 KEGG pathway enrichment analysis identified “Protein processing in the endoplasmic reticulum” (map04141) as significantly enriched in the comparison between healthy and insulin-resistant cells (adjusted *p*-value = 0.0016). This pathway includes 16 proteins from the dataset out of 132 genes in the pathway, indicating a strong overrepresentation. Proteins contributing to this enrichment include Protein disulfide-isomerase (P07237, P13667, P51571, P51668, Q15084), Endoplasmic reticulum chaperone BiP (P11021), Endoplasmin (P14625), Calreticulin (P27797), Translocon-associated proteins (P30101, Q9NR34), Ubiquitin-conjugating enzyme E2 D1 (P60059), Protein transport Sec61 subunit gamma (Q13217), DnaJ homolog C member 3 (Q13438), Protein OS-9 (Q15629), Translocating chain-associated membrane protein 1 (Q58FF3), Putative endoplasmin-like protein (Q9Y4L1), Mannosyl-oligosaccharide 1,2-alpha-mannosidase IC (Q9Y4L1), and Hypoxia up-regulated protein 1 (Q9Y4L1).

The enrichment of this pathway suggests that insulin-resistant cells exhibit significant alterations in ER protein folding, quality control, and stress responses, which is consistent with previous findings linking ER stress to insulin resistance. Key molecular functions affected include protein folding, disulfide bond formation, and chaperone-mediated quality control, highlighting the potential accumulation of misfolded proteins and activation of the unfolded protein response (UPR) in insulin-resistant states. Overall, these results indicate that insulin resistance is associated with dysregulated ER protein processing, which could contribute to cellular stress and impaired insulin signaling.

KEGG pathway enrichment analysis comparing insulin-resistant HepG2 cells with those treated with the peptide revealed a significant impact on mitochondrial and neurodegeneration-associated pathways. The top enriched pathway was oxidative phosphorylation (KEGG pathway: MAP00190), with key proteins including NADH dehydrogenase [ubiquinone] 1 beta subcomplex subunit 4 and cytochrome c oxidase subunit 3, indicating significant modulation of mitochondrial electron transport chain components [17]. in response to the peptide restoring ATP production and reducing mitochondrial dysfunction commonly observed in insulin-resistant hepatocytes. Other enriched pathways included Parkinson’s disease (MAP05012; adjusted *p*-value = 0.0021), driven by NADH dehydrogenase 1 beta subcomplex subunit 4, Cytochrome c oxidase subunit 3, and Guanine nucleotide-binding protein G(i) subunit alpha-1, and Alzheimer’s disease (MAP05010; adjusted *p*-value = 0.011), anchored by NADH dehydrogenase 1 beta subcomplex subunit 4, Cytochrome c oxidase subunit 3, Cyclic AMP-dependent transcription factor ATF-6 alpha, and Presenilin-1. While these pathways are classically associated with neurons, the proteins involved are ubiquitously expressed and functional in hepatocytes, particularly in regulating mitochondrial respiration, oxidative stress, and ER-mitochondrial crosstalk. Their enrichment suggests that the peptide not only restores energy metabolism but may also improve proteostasis and cellular stress responses, which are critical for reversing insulin resistance in liver cells.

Collectively, these findings indicate that the peptide’s therapeutic effects are mediated through enhancement of oxidative phosphorylation, stabilization of mitochondrial function, and modulation of stress-responsive proteins. The overlap of these proteins across multiple pathways underscores their central role in maintaining hepatocyte metabolic homeostasis, providing a mechanistic link between improved mitochondrial function and peptide-induced alleviation of insulin resistance.

Globally, these results indicate that the peptide’s anti-diabetic effects in HepG2 cells are likely mediated through restoration of oxidative phosphorylation and mitochondrial homeostasis, which are central to reversing insulin resistance, rather than implying that hepatocytes develop neurodegenerative diseases.

#### 3.3.4. Domain Enrichment Analysis

##### Healthy Cells (Control) vs. Insulin Resistant Cells (MOD): Domain Enrichment in Insulin Resistance

**Figure 6:**
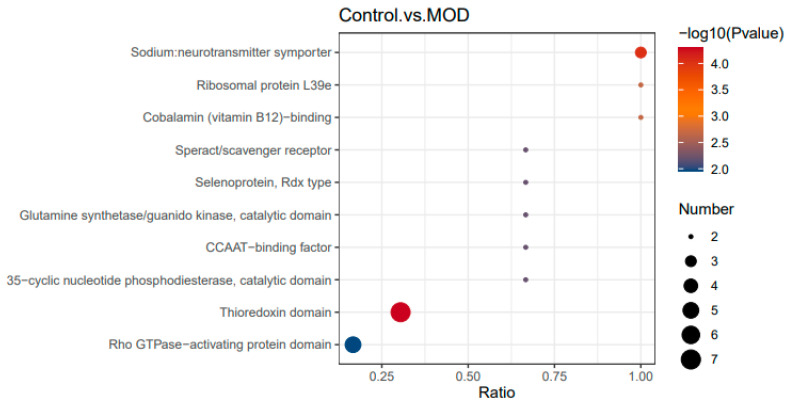
Domain enrichment analysis of healthy versus insulin-resistant cells. Differentially expressed proteins identified from proteomic profiling of healthy cells and insulin-resistant cells were subjected to InterPro (IPRT) domain enrichment analysis. The x-axis represents the enrichment ratio of proteins associated with each InterPro domain, while the y-axis lists the enriched functional domains. Bubble size indicates the number of proteins contributing to each domain, and color intensity corresponds to −log10 (*p*-value). Statistical significance was assessed using adjusted *p*-values corrected for multiple testing (Benjamini–Hochberg method), with an adjusted *p* < 0.05 considered significant. Domains with adjusted *p* ≥ 0.05 are shown as non-significant enrichment trends.

The IPRT domain enrichment analysis (see Figure 6) comparing healthy and insulin-resistant cells identified two protein domains that were significantly enriched based on the adjusted *p*-value threshold (<0.05), indicating biologically meaningful alterations associated with insulin resistance. The Thioredoxin domain (IPR013766, adjusted *p* = 0.021457) was significantly altered, with Protein disulfide-isomerase (PDI; anchor protein: P07237) emerging as a central contributor alongside other thioredoxin domain-containing proteins (PDIA3, PDIA4, PDIA6, TXNDC9, TXNDC15, TXNDC16). This enrichment points to a pronounced disruption in redox homeostasis, protein folding, and endoplasmic reticulum (ER) stress regulation in insulin-resistant cells, processes well known to interfere with insulin signaling and metabolic homeostasis. The prominence of PDI as an anchor protein suggests that altered oxidative protein folding and ER stress responses are key molecular features distinguishing insulin-resistant cells from healthy counterparts.

In parallel, the sodium: neurotransmitter symporter domain (IPR000175, adjusted *p* = 0.021457) was also significantly enriched, with the sodium- and chloride-dependent taurine transporter (anchor protein: P31641) representing a major node, together with creatine transporter 1 (P48029) and glycine transporter 1 (P48067). This finding indicates that insulin resistance is associated with changes in membrane transport systems involved in osmolyte balance, amino acid handling, and cellular energetics. Taurine and creatine transport are closely linked to mitochondrial function and glucose metabolism, suggesting that impaired substrate transport contributes to the metabolic inflexibility observed in insulin-resistant cells.

In contrast, all other identified domains including ribosomal protein L39e, cobalamin-binding enzymes, scavenger receptor related proteins, cyclic nucleotide phosphodiesterase’s, transcriptional regulators, selenoproteins, and Rho GTPase-activating proteins displayed adjusted *p* values > 0.05 and therefore did not reach statistical significance. While these domains may still participate in insulin resistance-associated pathways, their changes were not sufficiently robust after multiple-testing correction. Therefore, the IPRT domain enrichment results emphasize that insulin resistance is primarily characterized by significant perturbations in redox/ER stress regulation and solute transport systems, rather than global alterations across all functional protein domains.

##### Domain Enrichment Analysis Between Peptide Treated Cells and Insulin Resistant Cells

**Figure 7:**
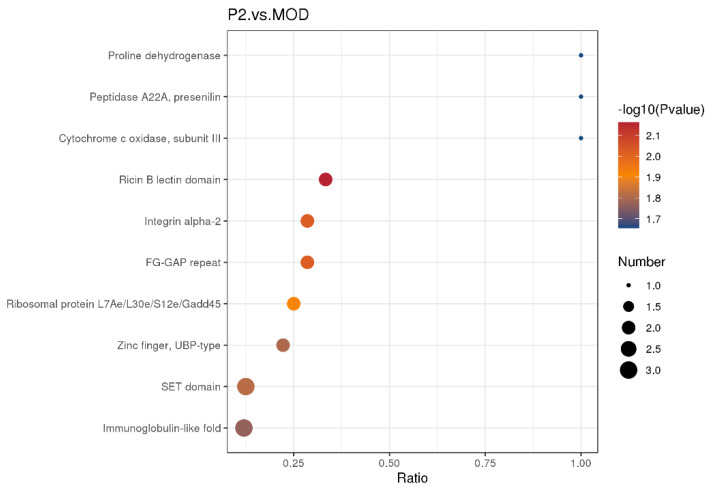
Domain enrichment analysis between insulin-resistant cells and insulin resistant cells treated with peptide (P2). Differentially expressed proteins identified from proteomic profiling of healthy cells and insulin-resistant cells were subjected to InterPro (IPRT) domain enrichment analysis. The x-axis represents the enrichment ratio of proteins associated with each domain, while the y-axis lists the enriched functional domains. Bubble size indicates the number of proteins contributing to each domain, and color intensity corresponds to −log10 (*p*-value). Statistical significance was assessed using adjusted *p*-values corrected for multiple testing (Benjamini–Hochberg method), with an adjusted *p* < 0.05 considered significant. Domains with adjusted *p* ≥ 0.05 are shown as non-significant enrichment trends.

The IPRT domain enrichment analysis comparing insulin-resistant cells with peptide-treated insulin-resistant cells (Figure 7) did not identify any protein domains reaching statistical significance, as all observed adjusted *p* values were 0.0948, exceeding the significance threshold of 0.05. This indicates that peptide treatment did not induce strong, domain-level proteomic remodeling after multiple-testing correction. Nevertheless, several biologically relevant trends emerged that suggest coordinated but moderate molecular shifts associated with peptide intervention.

Notably, domains linked to cell–matrix interaction and signaling, including the FG-GAP repeat (IPR013517) and integrin alpha-related domains (IPR013649), were represented by Integrin alpha-L (P20701) and Integrin alpha-3 (P26006) as anchor proteins. Although not statistically significant, changes in integrin-associated domains suggest that peptide treatment may influence cell adhesion, cytoskeletal organization, and outside-in signaling, processes closely connected to insulin sensitivity and cellular stress responses.

Several domains involved in epigenetic and post-translational regulation also appeared, such as the SET domain (IPR001214) with histone methyltransferases SUV39H1 (O43463), NSD3 (Q9BZ95), and KMT2B (Q9UMN6) serving as anchor proteins. In parallel, the UBP-type zinc finger domain (IPR001607), represented by USP22 (Q9UPT9) and USP16 (Q9Y5T5), points toward altered ubiquitin-mediated protein turnover. These trends imply that peptide treatment may subtly modulate chromatin state and protein stability in insulin-resistant cells, potentially contributing to longer-term transcriptional reprogramming rather than acute proteomic shifts.

Metabolic and mitochondrial-related domains were also detected, including Cytochrome c oxidase subunit III (IPR000298; anchor protein P00414), Proline dehydrogenase (IPR002872; O43272), and arginine methyltransferase NDUFAF7 (IPR003788; Q7L592). Although not significant, their presence suggests a tendency toward mitochondrial functional adjustment and amino acid metabolism remodeling following peptide exposure processes highly relevant to insulin resistance and energy homeostasis.

Additionally, domains related to stress response and growth control, such as GADD45 alpha (P24522) within the Ribosomal protein L7Ae/L30e/S12e/Gadd45 domain (IPR004038), and signaling-associated domains like the immunoglobulin-like fold (IPR013783) and presenilin peptidase domain (IPR001108), hint at peptide-induced modulation of inflammatory signaling, cell survival, and membrane-associated proteolysis.

In summary, while no IPR titles achieved statistical significance (adjusted *p* < 0.05), the observed domain patterns suggest that peptide treatment of insulin-resistant cells may exert subtle, coordinated effects on integrin signaling, epigenetic regulation, mitochondrial metabolism, and stress-response pathways. These changes may precede or complement the more pronounced signaling and functional improvements observed at the pathway or phenotypic level rather than at broad proteomic domain enrichment.

#### 3.3.5. Subcellular Localization Analysis of Differential Proteins

**Figure 8:**
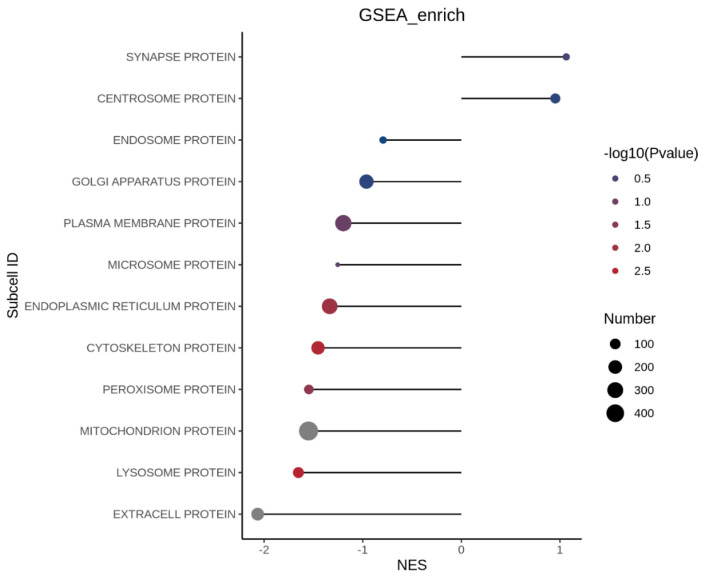
Subcellular gene set enrichment analysis (GSEA) between healthy cells and insulin-resistant HepG2 cells. Gene Set Enrichment Analysis (GSEA) was performed to compare protein localization between healthy cells and insulin-resistant HepG2 cells using a ranked list of all quantified proteins. Enrichment was assessed against Gene Ontology (GO) gene sets, and results are presented as normalized enrichment scores (NES). Positive NES values indicate gene sets enriched in peptide-treated cells, whereas negative NES values indicate enrichment in untreated insulin-resistant cells. Dot size represents the number of proteins contributing to each GO term, and dot color corresponds to −log10 (*p* value). Only gene sets meeting the statistical threshold (nominal *p* < 0.05 and FDR-adjusted q < 0.25, unless otherwise stated) are shown.

**Figure 9:**
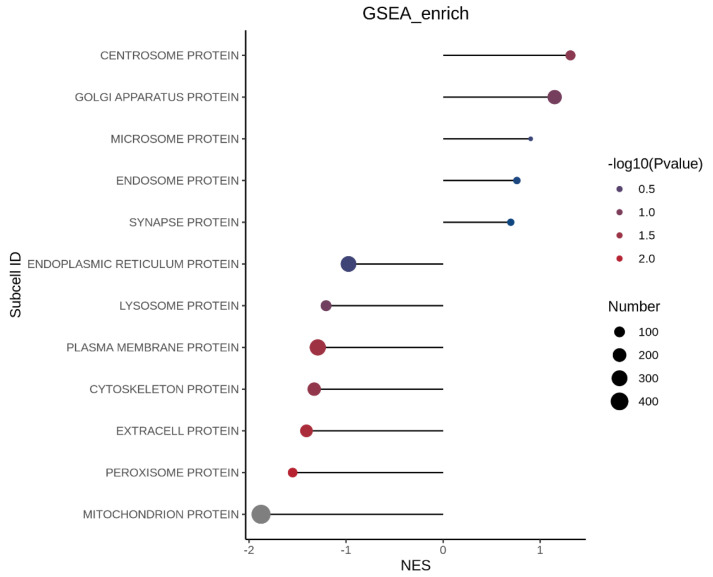
Subcellular gene set enrichment analysis (GSEA) between insulin-resistant HepG2 cells (MOD) and insulin-resistant HepG2 cells treated with peptide (P2). Gene Set Enrichment Analysis (GSEA) was performed to compare protein localization between healthy cells and insulin-resistant HepG2 cells using a ranked list of all quantified proteins. Enrichment was assessed against Gene Ontology (GO) gene sets, and results are presented as normalized enrichment scores (NES). Positive NES values indicate gene sets enriched in peptide-treated cells, whereas negative NES values indicate enrichment in untreated insulin-resistant cells. Dot size represents the number of proteins contributing to each GO term, and dot color corresponds to −log10 (*p* value). Only gene sets meeting the statistical threshold (nominal *p* < 0.05 and FDR-adjusted q < 0.25, unless otherwise stated) are shown.

The subcellular localization of differentially expressed proteins (DEPs) was analyzed to understand how insulin resistance (MOD) and peptide treatment (P2) influence protein distribution within the cell. Figure 8 and Figure 9 presents this analysis for each comparison.

##### Protein Localization Between Healthy Cells and Insulin Resistant Cells

The lysosomal protein compartment showed significant alteration between healthy and insulin-resistant cells (adjusted *p* = 0.0219), anchored by proteins central to lipid trafficking, sphingolipid metabolism, and membrane turnover. The presence of NPC intracellular cholesterol transporter 1 (NPC1) highlights disrupted lysosomal cholesterol efflux, a process closely linked to impaired insulin signaling and lipid accumulation in insulin-resistant states. Acid ceramidase, a key regulator of ceramide catabolism, suggests dysregulated sphingolipid metabolism, consistent with elevated ceramide levels known to inhibit insulin receptor and AKT phosphorylation. Palmitoyl-protein thioesterase 1 points to altered depalmitoylation and protein recycling within lysosomes, potentially affecting membrane-associated signaling proteins. In addition, CD44 antigen, a multifunctional receptor involved in inflammation and extracellular matrix interactions, indicates a link between lysosomal remodeling and inflammatory signaling. Collectively, these anchor proteins suggest that lysosomal dysfunction in insulin-resistant cells contributes to lipid overload, defective autophagic flux, and inflammatory signal amplification.

The extracellular protein compartment exhibited the most pronounced and statistically robust alteration (adjusted *p* = 0), underscoring extensive remodeling of the secretory and extracellular matrix environment in insulin resistance. This compartment was anchored by proteins involved in lipid transport, growth factor signaling, matrix organization, and immune modulation. The identification of apolipoprotein B-100 reflects altered lipoprotein handling and extracellular lipid burden, a hallmark of metabolic dysregulation. Growth and signaling mediators such as platelet-derived growth factor subunit A (PDGF-A) and angiopoietin-related protein 2 indicate enhanced paracrine signaling that may promote inflammation, fibrosis, and insulin resistance. Structural matrix components including collagen alpha-1 (VIII) chain, fibrillin-1, and lysyl oxidase homolog 3 suggest extracellular matrix stiffening and remodeling, processes known to impair insulin sensitivity through mechano transduction pathways.

Several extracellular chaperones and immune-modulatory proteins further emphasize a stress-adaptive secretory phenotype. Clusterin and calreticulin are associated with protein quality control and cellular stress responses, while galectin-3-binding protein and lactadherin are linked to immune activation and macrophage recruitment. The presence of ubiquitin-like protein ISG15 indicates activation of interferon-related inflammatory pathways. Additionally, sex hormone-binding globulin and mesencephalic astrocyte-derived neurotrophic factor (MANF) point toward altered endocrine and stress-response signaling beyond the intracellular space. Finally, prenylcysteine oxidase-like protein suggests changes in extracellular protein turnover and redox regulation.

Within the mitochondrial protein compartment, several anchor proteins underpin the significant alteration observed between healthy and insulin-resistant cells (adjusted *p* = 0.0347). Proteins directly involved in mitochondrial energy metabolism were prominently represented, including NADH-ubiquinone oxidoreductase chain 3, pyruvate dehydrogenase kinase isozyme 2 (PDK2), methylmalonyl-CoA mutase, and methylmalonate-semialdehyde dehydrogenase, indicating impaired oxidative metabolism and altered substrate utilization in insulin resistance. The presence of proline dehydrogenase 1 and sulfite oxidase further suggests dysregulation of amino acid catabolism and redox balance. Importantly, stress-responsive and protective mitochondrial proteins such as ferritin heavy chain and ferroptosis suppressor protein 1 (FSP1) highlight enhanced oxidative stress and lipid peroxidation defense mechanisms. The identification of Rab-7L1, StAR-related lipid transfer protein 13, and sphingosine kinase 2 links mitochondrial dysfunction to vesicular trafficking and lipid signaling, processes increasingly recognized as modulators of insulin sensitivity. Notably, the detection of ER chaperone BiP and DNAJ homolog subfamily C member 3 within this group suggests crosstalk between mitochondrial stress and the unfolded protein response, reinforcing the concept of organelle stress coupling in insulin resistance. Alterations in the peroxisomal protein compartment (adjusted *p* = 0.0267) were anchored by proteins essential for peroxisome biogenesis, membrane integrity, and organelle dynamics. Peroxisomal membrane protein PEX13 emerged as a key structural component, indicating potential impairment in peroxisomal protein import. The presence of WD repeat-containing protein 81 suggests disruptions in endosomal–peroxisomal interactions and intracellular trafficking. Additionally, mitochondrial fission 1 protein (Fis1) highlights a functional link between peroxisomes and mitochondria, supporting coordinated regulation of lipid oxidation and reactive oxygen species metabolism. Together, these anchor proteins point toward compromised fatty acid handling and redox homeostasis, both of which contribute to metabolic inflexibility in insulin-resistant cells.

The cytoskeletal protein category (adjusted *p* = 0.0443) was characterized by anchor proteins involved in structural organization, signal scaffolding, and ubiquitin-mediated regulation. Proteins such as Advillin and Shroom2 indicate remodeling of actin and cytoskeletal architecture, which is critical for insulin receptor localization and glucose transporter trafficking. Multiple WD repeat proteins (WDR43, WDR47, WDR9) suggest altered protein–protein interaction networks and scaffolding functions. Regulatory proteins including LIM domain-containing protein Ajuba and BTB/POZ domain-containing protein 1 are known modulators of mechanotransduction and signaling pathways, potentially influencing insulin signal propagation. Furthermore, the identification of probable E3 ubiquitin-protein ligase MID2 points to enhanced ubiquitin-dependent turnover of cytoskeletal or signaling proteins, which may impair insulin responsiveness by destabilizing key signaling complexes.

Taken together, the lysosomal and extracellular anchor proteins reveal that insulin resistance is accompanied by a coordinated disruption of intracellular lipid handling and extracellular signaling environments. Lysosomal dysfunction promotes lipid and ceramide accumulation, while extracellular matrix and secreted factor remodeling amplify inflammatory and metabolic stress signals, reinforcing insulin resistance at both cellular and tissue-mimetic levels.

##### Protein Localization Between Insulin Resistant Cells and Peptide Treated Insulin Resistant Cells

Subcellular GSEA revealed that peptide P2 treatment induced a pronounced reprogramming of organelle-specific protein signatures in insulin-resistant cells, with the most significant enrichment observed in the mitochondrial protein compartment (adjusted *p* = 0.00226, size = 485). This enrichment was anchored by proteins governing mitochondrial metabolism, redox balance, and stress adaptation, including NADH-ubiquinone oxidoreductase chain 3, proline dehydrogenase 1, methylmalonyl-CoA mutase, and methylmalonate-semialdehyde dehydrogenase, indicating restoration of oxidative and amino acid metabolism following P2 treatment. The presence of pyruvate dehydrogenase kinase isozyme 2 (PDK2) suggests modulation of glucose-derived carbon entry into the TCA cycle, consistent with improved metabolic flexibility. Stress-responsive proteins such as ferritin heavy chain and ferroptosis suppressor protein 1 point to enhanced control of iron-dependent lipid peroxidation and oxidative stress. Additionally, Rab-7L1, multidrug resistance-associated protein 1, and ER chaperone BiP highlight improved mitochondrial–vesicular trafficking and organelle stress crosstalk, supporting mitochondrial functional recovery as a primary target of P2 action.

The peroxisomal protein compartment also displayed significant enrichment following peptide treatment (adjusted *p* = 0.03085, size = 73), suggesting coordinated regulation of lipid oxidation and organelle dynamics. Anchor proteins such as peroxisomal membrane protein PEX13 indicate improved peroxisomal protein import and biogenesis, while WD repeat-containing protein 81 implies enhanced intracellular trafficking and organelle communication. The detection of mitochondrial fission 1 protein (Fis1) underscores the functional coupling between peroxisomes and mitochondria, particularly in fatty acid β-oxidation and ROS regulation. Furthermore, sphingosine kinase 2 and StAR-related lipid transfer protein 13 suggest normalization of sphingolipid signaling and intracellular lipid transport, pathways that are commonly dysregulated in insulin resistance.

In contrast, the extracellular protein compartment showed a moderate but non-significant trend toward enrichment following P2 treatment (adjusted *p* = 0.06957, size = 162). Anchor proteins including apolipoprotein B-100, sex hormone-binding globulin, and angiopoietin-related protein 2 suggest partial modulation of lipid transport and endocrine signaling. Growth and matrix-associated factors such as platelet-derived growth factor subunit A, collagen alpha-1 (VIII) chain, fibrillin-1, and lysyl oxidase homolog 3 indicate attenuation but not complete normalization of extracellular matrix remodeling. The presence of immune and stress-associated proteins (ISG15, clusterin, galectin-3-binding protein, lactadherin) suggests that peptide P2 may reduce, but not fully suppress, inflammation-associated secretory programs in insulin-resistant cells.

Other subcellular compartments showed no statistically significant enrichment following P2 treatment, including the cytoskeletal proteins (adjusted *p* = 0.09641; size = 190), plasma membrane proteins (adjusted *p* = 0.10138; size = 318), and lysosomal proteins (adjusted *p* = 0.15750; size = 105). This suggests that peptide P2 primarily exerts its effects through metabolic organelle recovery rather than broad structural remodeling or membrane trafficking changes. Similarly, the endoplasmic reticulum (adjusted *p* = 0.51193), centrosome (adjusted *p* = 0.32134), Golgi apparatus (adjusted *p* = 0.49371), microsome, endosome, and synapse-associated compartments (all adjusted *p* ≥ 0.96) remained largely unchanged, indicating preservation of basal cellular architecture.

Globally, this subcellular GSEA demonstrates that peptide P2 selectively reverses insulin resistance associated dysfunction by targeting mitochondrial and peroxisomal metabolic networks, with secondary effects on extracellular signaling. These findings strongly support a mechanism in which P2 restores insulin sensitivity through organelle-centric metabolic reprogramming rather than global proteome restructuring, aligning well with improved mitochondrial efficiency and lipid handling observed in peptide-treated insulin-resistant cells.

## 4. Conclusions

In summary, this DIA-based quantitative proteomic study defines insulin resistance in HepG2 cells as a state marked by coordinated alterations in protein abundance across redox-regulatory systems, endoplasmic reticulum protein processing machinery, and multiple organelle-associated networks. Enrichment analyses consistently highlighted dysregulation of the thioredoxin/protein disulfide isomerase system, ER-associated protein folding pathways, and mitochondrial, peroxisomal, and lysosomal proteomes, indicating widespread perturbation of intracellular protein homeostasis rather than isolated pathway defects.

Peptide P2 treatment did not induce global proteomic reversion but was associated with a selective reorganization of protein networks, most prominently within mitochondrial and peroxisomal compartments, as well as extracellular and secretory protein groups. These changes were reflected at the level of protein localization, domain composition, and pathway enrichment, suggesting a shift in organelle-linked proteomic architecture rather than direct evidence of restored signaling activity or metabolic function. Taken together, these findings demonstrate that insulin resistance and peptide responsiveness can be distinguished at the proteome-wide organizational level. This study provides a structural and systems-level proteomic framework for understanding how peptide exposure reshapes organelle-associated protein networks in insulin-resistant cells, establishing a foundation for future functional validation using targeted biochemical and cellular assays. These data position organelle-resolved proteomic remodeling as a sensitive molecular readout for peptide–cell interactions in insulin-resistant models.

## Data Availability

The original contributions presented in this study are included in the article. Further inquiries can be directed to the corresponding author.
